# Parechovirus A Detection by a Comprehensive Approach in a Clinical Laboratory

**DOI:** 10.3390/v10120711

**Published:** 2018-12-12

**Authors:** Bao-Chen Chen, Jenn-Tzong Chang, Tsi-Shu Huang, Jih-Jung Chen, Yao-Shen Chen, Ming-Wei Jan, Tsung-Hsien Chang

**Affiliations:** 1 Department of Microbiology, Kaohsiung Veterans General Hospital, Kaohsiung81362, Taiwan; metapneumonvirus83@gmail.com (B.-C.C.); tshuang@vghks.gov.tw (T.-S.H.); 2 Department of Pediatrics, Kaohsiung Veterans General Hospital, Kaohsiung 81362, Taiwan; jtchang109@yahoo.com.tw; 3 Faculty of Pharmacy, School of Pharmaceutical Sciences, National Yang-Ming University, Taipei 112, Taiwan; chenjj@ym.edu.tw; 4 Department of Medical Research, China Medical University Hospital, China Medical University, Taichung 404, Taiwan; 5 Department of Infectious Diseases, Kaohsiung Veterans General Hospital, Kaohsiung 81362, Taiwan; 6 Department of Medical Education and Research, Kaohsiung Veterans General Hospital, Kaohsiung 81362, Taiwan; davidjan0429@gmail.com; 7 Department of Medical Laboratory Science and Biotechnology, Chung Hwa University of Medical Technology, Tainan 717, Taiwan

**Keywords:** human parechovirus, immunofluorescence assay, virus diagnosis, clinical laboratory, emerging virus

## Abstract

Parechovirus A (Human parechovirus, HPeV) causes symptoms ranging from severe neonatal infection to mild gastrointestinal and respiratory disease. Use of molecular approaches with RT-PCR and genotyping has improved the detection rate of HPeV. Conventional methods, such as viral culture and immunofluorescence assay, together with molecular methods facilitate comprehensive viral diagnosis. To establish the HPeV immunofluorescence assay, an antibody against HPeV capsid protein VP0 was generated by using antigenic epitope prediction data. The specificity of the anti-HPeV VP0 antibody was demonstrated on immunofluorescence assay, showing that this antibody was specific for HPeV but not enteroviruses. A total of 74 HPeV isolates, 7 non–polio-enteroviruses and 12 HPeV negative cell culture supernatant were used for evaluating the efficiency of the anti-HPeV VP0 antibody. The sensitivity of HPeV detection by the anti-HPeV VP0 antibody was consistent with 5′untranslated region (UTR) RT-PCR analysis. This study established comprehensive methods for HPeV detection that include viral culture and observation of cytopathic effect, immunofluorescence assay, RT-PCR and genotyping. The methods were incorporated into our routine clinical practice for viral diagnosis. In conclusion, this study established a protocol for enterovirus and HPeV virus identification that combines conventional and molecular methods and would be beneficial for HPeV diagnosis.

## 1. Introduction

Parechovirus A (Human parechovirus, HPeV) belongs to the Picornaviridae, genus parechovirus. HPeV is a non-enveloped, single-stranded, positive-sense RNA virus [1]. Two species of parechovirus are classified: parechovirus A and B (formerly Ljungan virus, LV). Flanked by a 5′untranslated region (UTR) and a 3′ UTR, the open reading frame of HPeV genome encodes a single polyprotein that is processed to three structural or capsid-encoding proteins (VP0, VP3 and VP1, encompassing P1) and seven nonstructural proteins (2A, 2B, 2C [P2]; 3A, 3B, 3C and 3D[P3]) [2]. The different genotypes could be identified on the basis of their complete genome or viral protein 1 (VP1) sequences. Nineteen parechovirus A genotypes (HPeV1 to HPeV19) and 4 parechovirus B (LV1 to LV4) have been discovered on the basis of their complete genome or VP1 sequences (http://www.picornastudygroup.com/types/parechovirus/parechovirus.htm).

HPeV1 and HPeV3 are the most common genotypes reported [3,4]. Most cases of HPeV infection are asymptomatic or cause mild diseases of the gastrointestinal and respiratory tracts in children [5,6]. However, more severe consequences can occur with HPeV3 infection because of its strong association with infections of the central nervous system and neonatal sepsis, which raised a development concern in HPeV-infected infants. The severe HPeV3 infections are almost exclusively restricted to infants younger than 3 months; it seems that hospital-acquired infection in a neonatal department plays an important role in HPeV infections [7,8,9,10,11,12,13,14].

The RT-PCR test is the main approach for HPeV detection [15,16,17,18]. The conventional methods of virus isolation are also important and widely established for virus diagnosis in the clinical microbial laboratory [19]. In certain cases, isolating a virus is needed to clarify the etiology of the disease and for further investigation [20]. Immunofluorescence assay (IFA) is a simple and rapid method, requiring relatively little hands-on time in a clinical laboratory setting [21]. Conducting an IFA requires a specific antibody. Antibodies are an important biological factor with various applications in the fields of therapeutics, diagnostics and research. A number of different methods are commonly used to evaluate the antigen–antibody interactions [22]. Antigen epitope scanning with a bioinformatics approach provides powerful tool to map the antigen epitope for further applications.

A simple conventional method for HPeV detection is required to compensate for the deficiency of molecular approaches. In this study, by scanning the antigen epitope in the VP0 polypeptide sequence, we found a hit sequence covering the previously identified antigenic region of VP0 [23]. An IFA was established to detect HPeV by using a rabbit polyclonal antibody to HPeV VP0 in cell cultures inoculated with specimens and was compared with RT-PCR. Furthermore, we also established an applicable comprehensive protocol for routine clinical practice for the diagnosis of HPeV and enterovirus infection.

## 2. Materials and Methods

### 2.1. Ethics Statement

This study was approved by the Institutional Review Board of Kaohsiung Veterans General Hospital (protocol no. VGHKS15-EM10-01, 15/08/2015) and conformed to the current ethical principles of the Declaration of Helsinki.

### 2.2. Virus and Cell Lines, Chemicals

HPeV subtypes1, 3, 4 and 6; Echovirus9, 11 and 71; coxsackievirus A2, A10, A16, B3 and B5; and Aichi virus were isolated from clinical specimens by the Virology Group, Department of Microbiology, Kaohsiung Veterans General Hospital. A549 cells (human lung epithelial carcinoma cells, ATCC: CCL-185, Manassas, VA) and Vero cells (*Chlorocebus sp.* kidney epithelial cells, ATCC: CCL-81) were cultured in Dulbecco’s modification of Eagle medium (DMEM) supplemented with 10% fetal bovine serum (FBS; ThermoFisher Scientific, Waltham, MA, USA). DBTRG-05MG human glioblastoma cells (BCRC: 60380, Hsinchu, Taiwan) were cultured in RPMI 1640 medium supplemented with 10% FBS (Thermo Fisher Scientific, Waltham, MA, USA).

### 2.3. Antibodies

The Pep VP0-21, a 21-amino-acid synthetic peptide, NLTQHPSAPTIPFTPDFRNVD, derived from the conserved VP0 caspid protein, was used for anti-HPeV1 VP0 antibody generation [24,25]. Twelve-week-old New Zealand white rabbits (Crl:KBL, NZW; Livestock Research Institute, Council of Agriculture, Taiwan) were primed with 0.5 µg Pep VP0-21 peptide in 100 µL DMSO plus 100 µL Freund’s complete adjuvant. After 20 days, the rabbits received nine boosts shots at 10-day intervals over 3 months. Seven days after the last boost, the sera were harvested by cardiac puncture in the anesthetized rabbit, and tested by dot blot analysis for antibodies to VP0 peptide. The anti-HPeV VP0 antibody was purified by using a VP0 peptide affinity column.

An amount of 1000, 100, 10 and 1 ng VP0 peptide was spotted on PVDF membranes (EMD Millipore, Burlington, MA, USA) and allowed to air-dry, and the unbound sites was blocked for 3 h in 3% bovine serum albumin (BSA) in phosphate buffered saline (PBS), the membrane was then incubated with anti-serum with dilution of various ranges, at 4 °C overnight. After the washes, the blots were placed in 1:1000 diluted peroxidase-conjugated anti-rabbit IgG antibody (Jackson Immuno Research Laboratory, West Grove, PA, USA) for 1 h at room temperature (RT) and washed again. The antigen–antibody complex was visualized by using 3,3′-diaminobenzidine (1 mg/mL, in 50 mM citrate buffer, pH 5.5; Sigma-Aldrich, St. Louis, MO, USA) as the chromogen.

### 2.4. Immunofluorescence Assay

We inoculated 2×10^5^ cells in 16 × 125 mm culture tubes (Corning, NY, USA) with specimens or mock control. Cells were scrapedfrom the culture tube and spotted onto PTFE diagnosticslide wells (ThermoFisher Scientific, Waltham, MA, USA). After an air dry process in a biosafety cabinet, the cells were fixed with cold acetone for 10 min at 4 °C. After twowashes with PBS, cells were blocked with 10% skim milk in PBS for 30 min at RT. Cells were incubated with anti-HPeV VP0 antibody (1:300 in PBS with 5% skim milk); then, Alexa Fluor-488 goat anti-rabbit IgGsecondary antibody (1:300, ThermoFisher Scientific, Waltham, MA, USA) along with Evans blue counterstaining (0.01~0.02%, Sigma-Aldrich, St. Louis, MO, USA), each for 1 h at 25 °C. The HPeV-infected cells showed green fluorescence, and the red fluorescence of Evans blue staining indicated the location of cells. The immunofluorescence signalswere observed with a Zeiss Axio Observer A1 microscope (Oberkochen, BW, Germany).

In the IFA test of antibody specificity, 1 × 10^5^ cells were grown in 12 well plates and then infected with different viruses for 24 h. The cells were fixed with 4% paraformaldehyde for 30 min at RT, and then permeabilized with 0.5% Triton X-100 for 10 min at RT before adding the anti-HPeV VP0 antibody. The DAPI staining indicated the nuclear location.

### 2.5. Immunoblotting Analysis

Mock or infected cells (1 × 10^5^) were lyzed with 100 µL Radioimmunoprecipitation assay buffer (RIPA) buffer (150 mM NaCl, 0.5% sodium deoxycholate, 1% NP40, 0.1% SDS, 50 mM Tris-HCl [pH 8.0]) containing 1× protease inhibitor and phosphatase inhibitor (Halt protease and phosphatase inhibitor single-use cocktail, ThermoFisher Scientific, Waltham, MA, USA). An amount of 100 µg cell extracts was separated by 10% SDS-PAGE and transferred to PVDF membranes, which were blocked with 10% skim milk in TBST buffer for 30 min at RT, incubated with anti-HPeV VP0 antibody (1:1000 in TBST with 5% skim milk) or anti-FLAG M2 antibody (1:5000 in TBST with 5% skim milk; Sigma-Aldrich, St. Louis, MO, USA) for 2 h at 25 °C. Then, horseradish peroxidase (HRP)-conjugated goat anti-rabbit or mouse IgGsecondary antibody (1:5000 in TBST with 5% skim milk; Jackson Immuno Research Laboratory) was added and incubated for 2 h at 25 °C. The chemiluminescence signals were visualized by using an enhanced chemiluminescence system (WesternBright ECl, Aadvansta Corp., Menlo Park, CA, USA). Images were acquired with a digital image system (UVP, LLC; Upland, CA, USA).

### 2.6. HPeV3 VP0 Expression Vector

Full-length cDNAs for HPeV1 VP0 (strain KVP6accessionno. KC769584) and HPeV3 VP0 (strain VGHKS-2007, accession no.KM986843) were cloned from A549 cells infected with HPeV1 and HPeV3 (MOI=5) for 24 h. First-strand cDNAs were synthesized from 5 µg total RNA prepared with Trizol (ThermoFisher Scientific, Waltham, MA, USA) and used to amplify full-length cDNA for the VP0 gene by conventional PCR. The HPeV1 and HPeV3 VP0 genes were cloned into the pCMV-3X FLAG tag vector (Sigma-Aldrich, St. Louis, MO, USA) with primers including restriction enzyme recognition sites as follows: HPeV1 VP0 forward-*Not*I: TT GCGGCCGCG ATGGAGACAATTAAGAGCATT; HPeV1 VP0 reverse-*Sal*I: GAG*TCGAC* TTA ATTGTCGTAAATACCAACGTC; HPeV3 VP0 forward-*Not*I: TTGCGGCCGCG ATG GAGTCTATCAAGGATTTAGTCAAT; and HPeV3 VP0 reverse-*Xba*I: CCTCTAGATTA ATTATCATAAATTTCCACATCTTC.

The accuracy of pCMV-3X-FLAG-HPeV1 VP0 or -HPeV3 VP0 plasmids was confirmed by restriction enzyme digestion, DNA electrophoresis analysis and nucleic acid sequencing. The pCMV-3X-FLAG-HPeV1 VP0 or -HPeV3 VP0 plasmid was prepared by using the CompactPrep Plasmid Midi Kit (Qiagen, Venlo, The Netherlands); 2 µg of each plasmid was transfected into Vero cells by using Turbofect transfection reagent (ThermoFisher Scientific, Waltham, MA, USA) to express FLAG-fused VP0 protein.

### 2.7. Laboratory Diagnosis

Virus identification involved the protocol for *Enterovirus* diagnosis of the Virology Group, Department of Microbiology, Kaohsiung Veterans General Hospital [15]. The clinical samples of rectal, throat swabs or cerebrospinal fluid were inoculated into five different cell lines, human normal lung diploid fibroblast (MRC-5), human rhabdomyosarcoma (RD), human lung adenocarcinoma (A549), rhesus monkey kidney (LLC-MK2), and Madin-Darby canine kidney (MDCK) cells in culture tubes. The viral cultures were incubated at 35 °C in a 5% CO_2_ atmosphere and observed twice a week for cytopathic effect (CPE) at 4× magnification for 21 days before they were reported as negative.

For specimens that showed a CPE, IFA was performed with pan-*enterovirus* antibody (Pan-EV, L66J, ThermoFisher Scientific, Waltham, MA, USA) for *Enterovirus* diagnosis and anti-HPeV VP0 antibody for HPeV diagnosis. RT-PCR analysis of the HPeV5′UTR and VP1 gene was conducted simultaneously for HPeV determination and typing with the following primer sets: 5′UTR-F1 (5′-CCACGCTYGTGGAYCTTATG-3′) and 5′UTR-R1 (5′-GGCCTT ACAACTAGTGTTTGC-3′); VP1-F1(5′-CCRRAAYTCRTGGGGTC-3′) and VP1-R1: (5′-TCYARYTGRTAYACAYKSTCTCC-3′). The PCR products were analyzed by electrophoresis on a 1.5% agarose gel, and then extracted for genotype identification by sequence analysis [8,15,26].

## 3. Results

### 3.1. VP0 Antigeneticity/Epitope Analysis and Antibody Production

**Figure 1:**
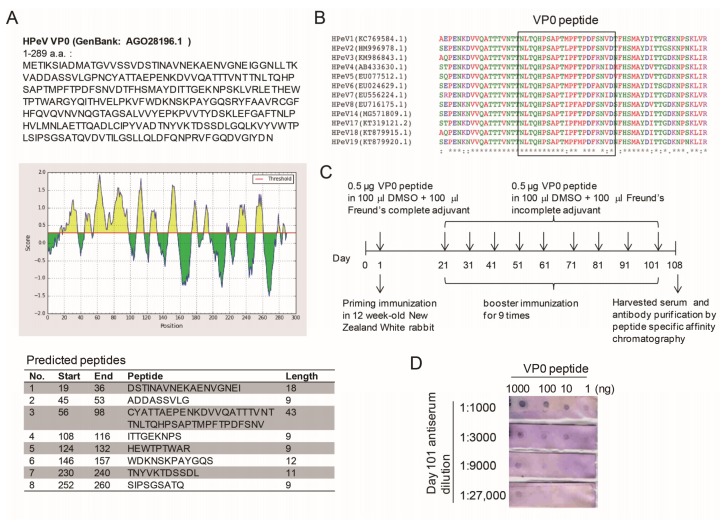
Epitope analysis and antibody production. (**A**) The 289 amino acid-polypeptide sequence of parechovirus A (Human parechovirus type 1, HPeV1) VP0 (Genbank: AGO28196.1) was used to predict an epitope by using the Bepipredlinear epitope prediction method (IEDB analysis resource, http://tools.immuneepitope.org/bcell/ or http://www.cbs.dtu.dk/services/BepiPred/). The eight-peptide sequence with antigenicity was shown. (**B**) Multiple sequence alignment of HPeV type 1-8, 14 and 17-19 VP0 peptides involved use of Clustal Omega, https://www.ebi.ac.uk/Tools/msa/clustalo/. The VP0 sequence of HPeV type 9-11 and 15-16 are not available in NCBI Genbank for analysis. (**C**) Schematicfor anti-HPeV VP0 antibody generation. (**D**) Antibody titration (dilution ranging 1:1000~1:27,000) of the anti-serum was measured by dot blotting with 1 to 1000 ng VP0 peptide spotted.

To establish a conventional method of IFA for HPeV identification, a specific antibody against HPeV was needed. The antigenic properties of HPeV1 indicated an antigenic site in the VP0 polypeptide [23]. Therefore, the HPeV1VP0 polypeptide sequence was scanned by using the Bepipredlinear epitope prediction method to identify the epitope for peptide synthesis and antibody production [27,28]. Eight peptide sequences were predicted (Figure 1A). As supported by previous reports [23,24] and the BLAST data, the No. 3 peptide sequence (amino acid position 56–98) contained a conserved region in the VP0 of HPeV type 1-8, 14 and 17-19 (Figure 1B). Thus, a 21-amino-acid synthetic VP-0 peptide, NLTQHPSAPTIPFTPDFRNVD, was used to vaccinate rabbits, followed by a total of 9 boosts over 3 months. At 7 days after the last boost, the rabbit was anesthetized for serum harvesting by cardiac puncture (Figure 1C). The dot blots of anti-serum against various amounts of VP0 peptide showed a titration range from 1:1000-27,000 (Figure 1D). After validation, the anti-HPeV VP0 antibody was further purified byVP0 peptide affinity chromatography.

### 3.2. Antibody Specificity Analysis by IFA and Immunoblotting

**Figure 2:**
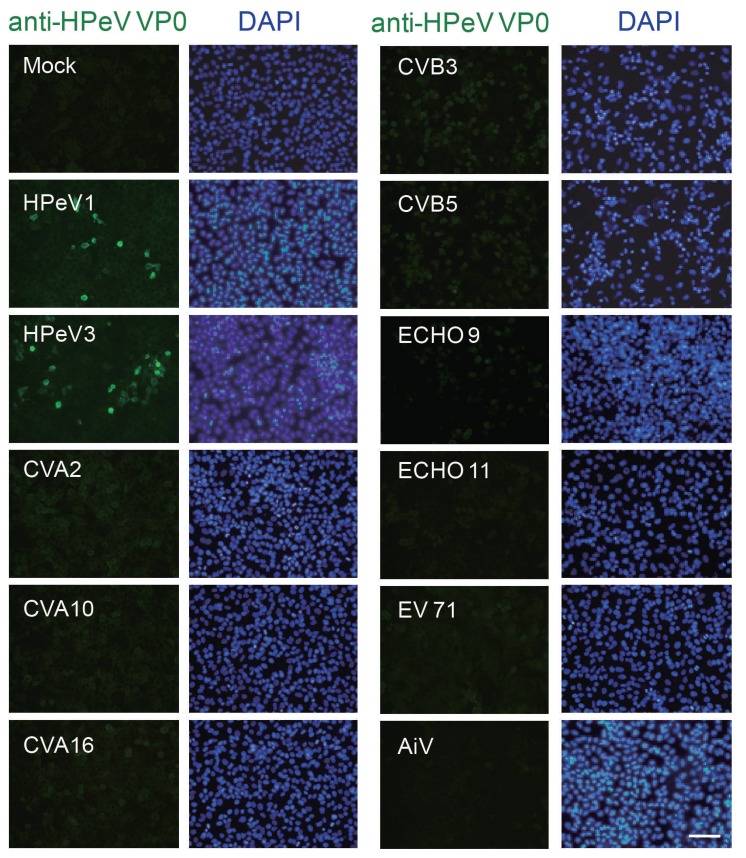
Specificity of anti-HPeV VP0 antibody. Immunofluorescence assay (IFA) was conducted in A549 cells (1 × 10^5^) infected with HPeV1, HPeV3, echovirus 9 (ECHO 9), ECHO 11, enterovirus 71 (EV71), coxsackievirus A2 (CVA2), CVA10 and CVA16, coxsackievirus B3 (CVB3) and CVB5, Aichi virus (AiV) and mock control for 24 h. Green fluorescence indicates viral-infected cells identified by anti-HPeV VP0 antibody. The DAPI staining indicated the nuclear location, scale bar: 100 μm.

A successful and applicable antibody relies on its specificity against antigen. To determine whether the anti-VP0 antibody specifically recognized HPeV VP0 protein but not other picornaviruses, A549 cells were infected with HPeV1, HPeV3 and picornaviruses such as echovirus 9 (ECHO 9) and 11; enterovirus 71 (EV71); coxsackievirus A2 (CVA2), A10 and A16; coxsackievirus B3 (CVB3) and B5; Aichi virus (AiV); and mock control. IFA was conducted after 24 h post infection (p.i.). The anti-HPeV VP0 specific antibody recognized cells infected with HPeV1 and HPeV3 but not other viruses (Figure 2).

**Figure 3:**
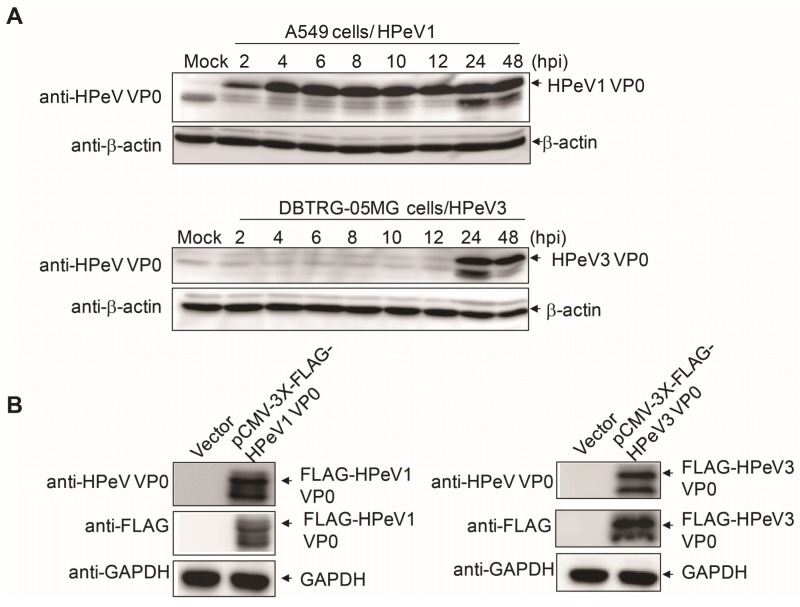
Immunoblotting assay with anti-HPeV VP0 antibody. (**A**) A549 cells (1 × 10^5^, upper panels) and DBTRG-05MG cells (1 × 10^5^, lower panels) were infected with HPeV1 and HPeV3 (MOI = 5), respectively. Cell lysates were harvested at various times post-infection (2 to 48 h) for VP0 detection by anti-HPeV VP0antibody. β-actin was a loading control. (**B**) pCMV-3X-FLAG-HPeV1 VP0 or -HPeV3 VP0plasmid (2 µg) was transfected into Vero cells (3 × 10^5^) for 30 h. The ectopic expression of FLAG-HPeV1 VP0 or -HPeV3 VP0 was detected by immunoblotting with anti-HPeV VP0 and anti-FLAG antibodies. GAPDH was a loading control.

The protein molecular weight of HPeV VP0 is estimated at about 32 kD. The immunoblotting analysis was conducted to confirm the anti-HPeV VP0 antibody detecting the correct size of protein in HPeV1- and HPeV3-infected A549 and DBTRD-05MG cells, respectively. HPeV1 VP0 expression was detected at the correct molecular size position as early as 2 h after infection; HPeV3 VP0 was detected at 24 and 48 h post-infection (Figure 3A). To further test the specificity of the anti-HPeV VP0 antibody, both HPeV1 VP0 and HPeV3 VP0 cDNA were cloned and inserted into a mammalian expression vector, pCMV-3X FLAG tag, to generate the FLAG-VP0 fusion protein. Immunoblotting with anti-VP0 antibody and also anti-FLAG antibody detected the expression of ectopic FLAG-HPeV1 VP0 and -HPeV3 VP0 proteins in Vero cells (Figure 3B). The full images of the immunoblots are shown in the Supplementary Figures S1 and S2. Together, these data validated the specificity of the anti-HPeV VP0 antibody.

### 3.3. HPeV Detection by RT-PCR and IFA

RT-PCR assay is the current method available for HPeV diagnosis. We compared the HPeV detection efficiency between RT-PCR and IFA. A total of 74 clinical HPeV isolates, seven non-polio/enterovirus (NPEV) isolates and 12 HPeV RT-PCR negative control samples were collected for testing in A549 cells. All 74 HPeV isolates with 5′UTR RT-PCR–positive were also IFA positive by staining for VP0 (Table 1). RT-PCR analysis of the VP1 gene involved 65 HPeV isolates; 16/65 samples (24.6%) were negative; this suggested that an additional primer set for VP1 RT-PCR was required for the HPeV isolates. None of the negative control isolates were detected with an anti-HPeV VP0 antibody. These data indicate a specific and consistent result of HPeV detection between 5′UTR RT-PCR and IFA (Table 1).

### 3.4. HPeV Diagnosis Protocol

**Figure 4:**
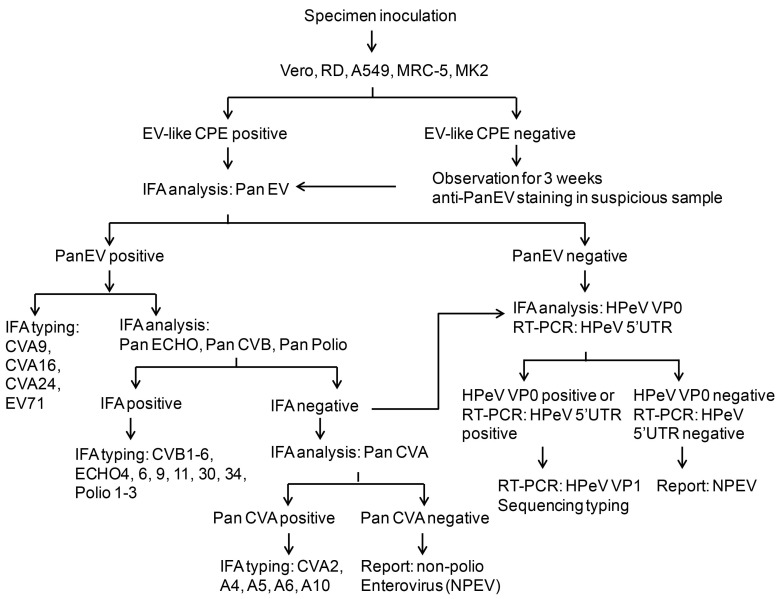
Schematic of HPeV diagnosis protocol. The schematic shows the process of HPeV diagnosis in a clinical virology laboratory. The procedures include specimen inoculation, cytopathic effect (CPE) evaluation, IFA staining and RT-PCR analysis of HPeV.

As shown in the above results, we established the new IFA method with anti-HPeV VP0 antibody. Therefore, the IFA was introduced into the routine diagnostic protocol for detecting HPeV or other picornaviruses in our clinical microbiology laboratory (Figure 4). The process begins with specimen inoculation in five different cell lines: Vero, RD, A549, MRC and MK2 cells. The CPE is observed every day for up to 3 weeks. During the observation period, the enterovirus antigen is directly detected by anti-Pan-EV antibody. The positive sample is subjected to identification of specific viruses including echovirus, CVA and CVB, enterovirus and poliovirus. The samples that are negative for anti-Pan-EV, anti-Pan-ECHO, anti-Pan-CVB or pan-polio virus antibody undergo anti-Pan-CVA staining for CVA infection or anti-HPeV VP0 staining along with 5′UTR RT-PCR analysis to identify HPeV infection. The HPeV subtype virus is confirmed by PCR amplification and sequencing the VP1 gene in a VP0 immunofluorescence- or 5′UTR-positive sample. The non-typeable virus would be reported as an NPEV. In brief, this protocol combines conventional and molecular methods to detect HPeV infection.

**Figure 5:**
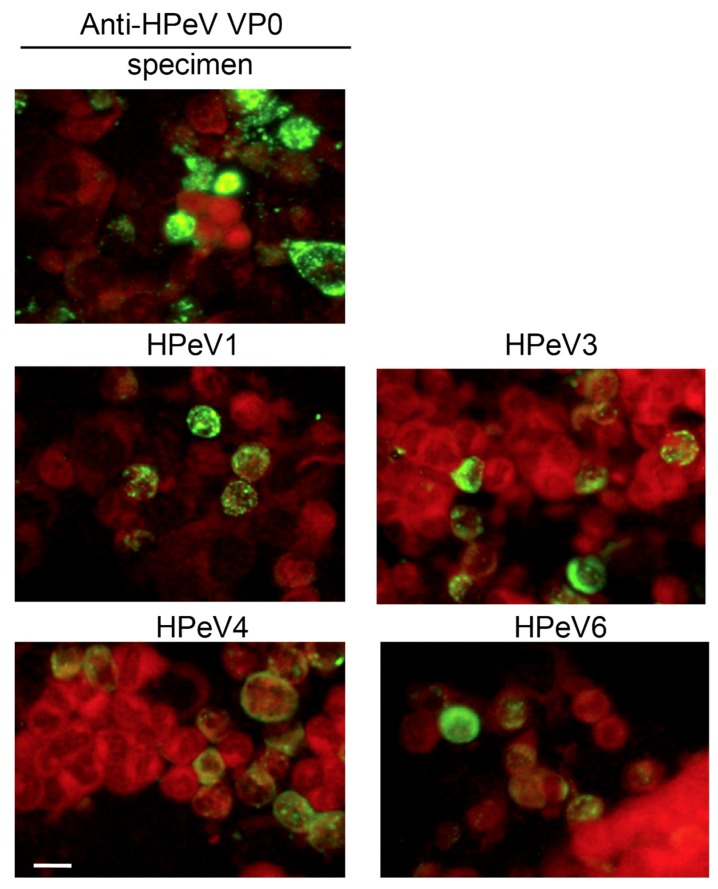
HPeV detection in clinical specimens from patients underwent HPeV diagnosis by IFA with anti-HPeV VP0 antibody. Green fluorescence indicates HPeV-infected cells (upper panel). Evans blue staining shows the cell location (red fluorescence). Cells infected with HPeV1, HPeV3 HPeV4 and HPeV6 were used as staining controls, scale bar: 20 µm.

The anti-HPeV VP0 antibody was used for HPeV diagnosis in samples of rectal or throat swabs or cerebrospinal fluid. Here, the HPeV1, HPeV3, HPeV4 and HPeV6 infections were tested along with specimen testing as the positive controls (Figure 5). The immunofluorescence signals in HPeV-infected cells indicated that the protocol was applicable in clinical practice.

## 4. Discussion

This study implemented the HPeV diagnosis procedure in a clinical virology laboratory with a conventional approach consisting of viral culture and IFA in addition to RT-PCR test. This protocol showed that the use of an antibody against the HPeVVP0 antigen was a good tool for detecting HPeV in cells. The sensitivity of HPeV detection by anti-HPeV VP0 antibody was consistent with HPeV 5′UTR RT-PCR finding (100%), and higher than that with VP1 RT-PCR (75.4%, Table 1). A comprehensive viral investigation can be established involving cell culture, CPE, IFA, virus isolation, RT-PCR and genotyping. This procedure has been included in our routine clinical practice of enterovirus diagnosis.

Conventional and molecular approaches are used in virus diagnosis; some are widely used. However, viral isolation in cell cultures is a routine method in specialized clinical laboratories. The isolated virus could be used as a control, complete investigations can be performed when needed, and representative clinical strains can be stored [20,29,30].

In Taiwan, when the virus identification in a clinical specimen is required, the specimen inoculation to different type of cells and IFA analysis would be conducted. This is the first-standard and routine method to diagnose viral infection in a clinical microbiology, and that relies on the fluorescence-conjugated antibody. For example, the LIGHT DIAGNOSTICS™ Pan-Enterovirus Reagent kit (MERCK) or other antibodies against the specific viruses would be used in enteroviruses diagnosis. Compared to the RT-PCR test, IFA is an easier method for a clinical medical technician in the clinical microbiology laboratory of a hospital. The IFA data will be reported to the Taiwan Center for Disease Control (Taiwan, CDC). In certain cases, the RT-PCR analysis will be conducted by Taiwan CDC to further investigate the virus types. This is the national system of enterovirus infection surveillance in Taiwan, which has been working for over a decade. The weekly report of enterovirus infection surveillance is available on the Taiwan CDC website. (https://nidss.cdc.gov.tw/en/Default.aspx?op=1).

Because the HPeV is an emerging virus in Taiwan, the clinical technicians need an easy and rapid method to identify this infection from clinical specimens. HPeV infection causes enterovirus-like CPE in cells; the IFA method could be applied to HPeV diagnosis. Thus, we established an incorporated routine-workflow for HPeV diagnosis including IFA and RT-PCR approaches in a clinical microbiology laboratory. The RT-PCR is an important method for virus typing; thus, RT-PCR is performed for HPeV typing in our protocol (Figure 4).

HPeV-1 capsid protein contained an important antigenic site in the N-terminal region of the VP0 protein (Figure 1). The peptide of this region, Pep VP0-21, a 21-amino-acid synthetic peptide, was also found to be an ELISA antigen in a seroprevalence survey [24]. The present data demonstrate that the antibody elicited by the Pep VP0-21 was able to specifically identify HPeV infection by using IFA, which showed that the antibody would be useful in HPeV diagnosis (Figure 2 and Figure 5). Moreover, the result suggested that epitope prediction would be a useful tool for antibody production without requiring a complete virus [16,31]. Furthermore, epitope prediction has shown its potential in vaccine development [32].

Seven epitope sequences throughout the HPeV1 full polypeptide were found in the database of Immune Epitope Database and Analysis resource (https://www.iedb.org/result_v3.php?cookie_id=fd38b5); the similar epitope sequence with PepVP0-21 is also in the list (Epitope ID: 65238). The information for other HPeV1 epitopes in the database may be useful for future analysis.

The efficiency of HPeV VP0 detection by antibody was evaluated by comparison with RT-PCR analysis (Table 1). The present data were consistent with the results of 5′-UTR RT-PCR; the detection rate was higher than that with VP1 RT-PCR (100% vs. 75.4%). We thought that when VP-1 RT-PCR amplification and sequencing is used for genotyping, specific genotype primers would be required. A similar comparison approach was used in the detection of the human metapneumovirus in a clinical laboratory. Their data showed 8% of the samples showed positive by using IFA and 21% showed positive with RT-PCR; direct IFA was not very sensitive (38%) under that clinical laboratory setting [21]. In our case, all the HPeV isolates could be identified by both 5′UTR RT-PCR and IFA. We thought that, along with the 5′UTR RT-PCR, IFA by staining HPeV VP0 would be an additional useful tool for HPeV diagnosis in a clinical laboratory.

The anti-HPeV0 antibody is also useful in the virology research of HPeV. The replication of HPeV1 and HPeV3 in vitro was previously measured by RT-PCR analysis [31]. Our data provide the VP0 protein level examination for HPeV investigation (Figure 3A).

Our data allowed us to establish a flow chart for HPeV and enterovirus diagnosis (Figure 4). These methods improve the HPeV diagnosis procedure in clinical practice. As shown in Figure 5, the IFA data for HPeV4 and HPeV6 were first revealed by using the anti-HPeV VP0 antibody.

Previously, the antisera against HPeV1 and HPeV3 were elicited by viral particle immunization in guinea pigs or rabbits for research in vitro [16,31]. However, the target epitopes by these antibodies were unknown, and the clinical application could not be assessed. Our study overcomes the impediments to HPeV diagnosis in clinical application by providing a specific antibody against HPeV VP0.

In conclusion, this study established a protocol for enterovirus and HPeV virus identification that combines conventional and molecular methods and would be beneficial for HPeV diagnosis.

## Figures and Tables

**Table 1:** The RT-PCR and immunofluorescence assay (IFA) results for human parechovirus (HPeV) detection.

Isolates	Total	RT-PCR: 5′UTR		IFA: Anti-VP0		RT-PCR: VP1		
		Pos. (n, %)	Neg. (n, %)	Pos. (n, %)	Neg. (n, %)	Pos. (n, %)	Neg. (n,%)	ND (n)
HPeV	74	74 (100)	0	74 (100)	0	49 (75.4) ^#^	16 (24.6) ^#^	9
HPeV negative control	12	0	12 (100)	0	12 (100)	0	12 (100)	0
NPEV	7	0	7 (100)	0	0	0	7 (100)	0

**#**: HPeV sample number of VP1 RT-PCR detection = 65. ND: non-detected. HPeV: RT-PCR positive of HPeV 5′UTR. HPeV negative control: HPeV negative cell culture supernatants (RT-PCR negative of HPeV 5′UTRor IFA negative of enteroviruses). NPEV: non-polio-enterovirus.

## Supplementary Materials

The following are available online at http://www.mdpi.com/1999-4915/10/12/711/s1, Figure S1: The full immunoblots images of Figure 3A. Figure S2: The full immunoblots images of Figure 3B.
