# Supplementary figures and images for: Parechovirus A Detection by a Comprehensive Approach in a Clinical Laboratory
Source: Viruses. 2018 Dec 12;10(12):711. doi: 10.3390/v10120711 (PMC6316871; doi:10.3390/v10120711)

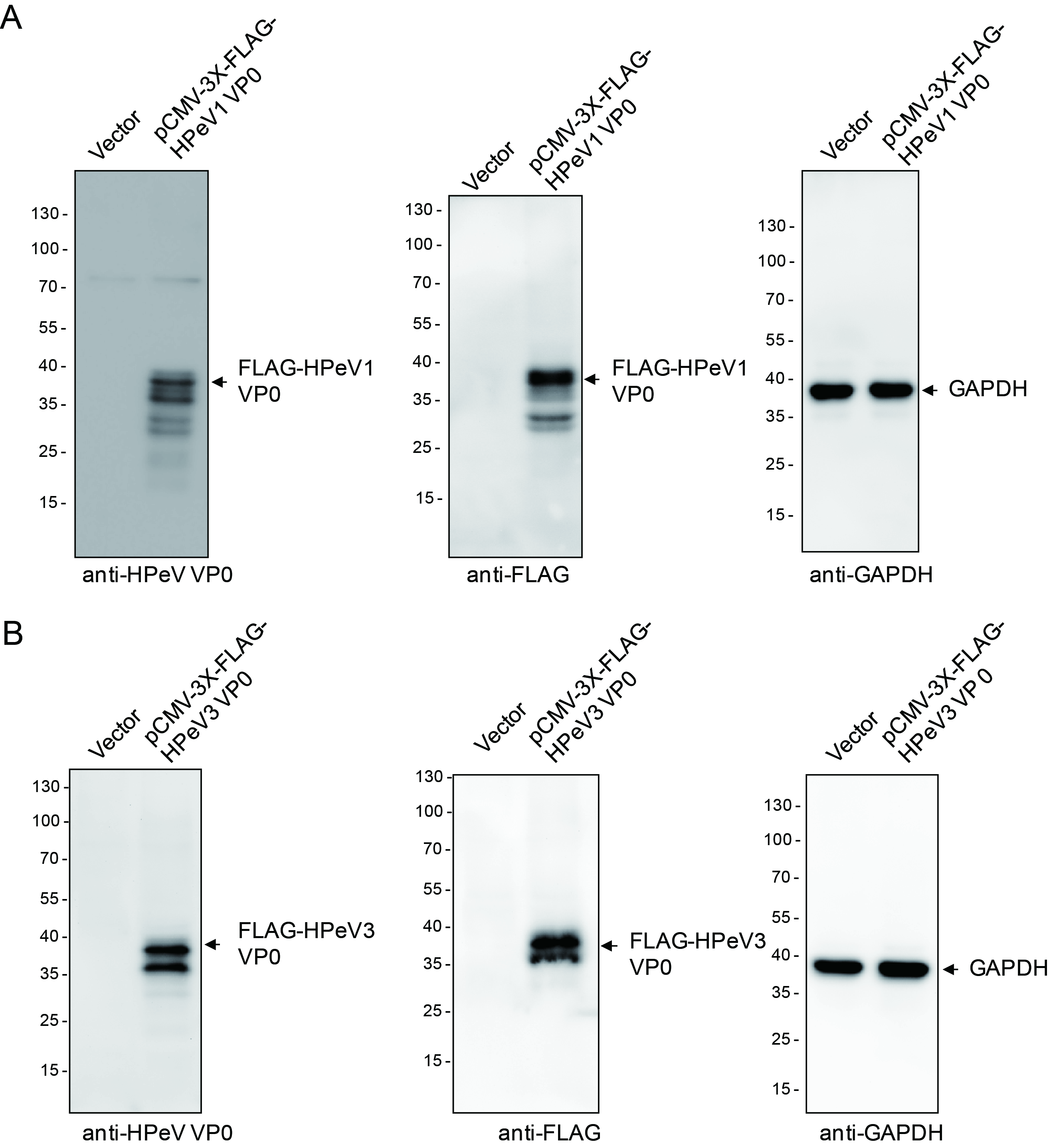

Supplement: Supplementary file 1 [file viruses-10-00711-s001.zip › Supplementary Figure 2.tif]

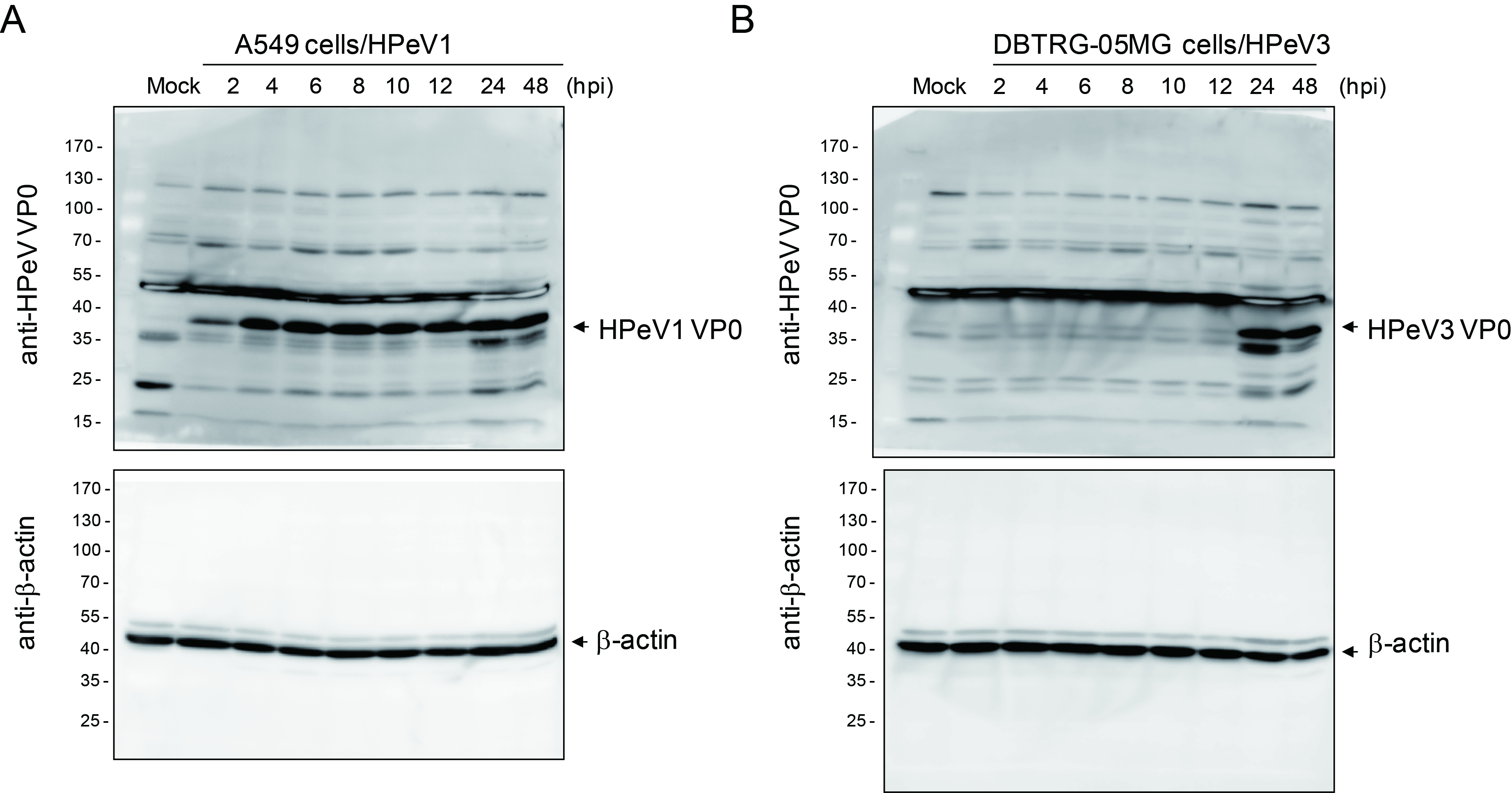

Supplement: Supplementary file 1 [file viruses-10-00711-s001.zip › Supplementary Figure 1.tif]
